# Typical and atypical presenting symptoms of breast cancer and their associations with diagnostic intervals: Evidence from a national audit of cancer diagnosis

**DOI:** 10.1016/j.canep.2017.04.010

**Published:** 2017-06

**Authors:** Minjoung Monica Koo, Christian von Wagner, Gary A. Abel, Sean McPhail, Greg P. Rubin, Georgios Lyratzopoulos

**Affiliations:** a University College London, 1-19 Torrington Place, London WC1E 6BT, UK; b University of Exeter, St Luke’s Campus, Heavitree Road, Exeter EX1 2LU, UK; c National Cancer Registration and Analysis Service, Public Health England Zone A, 2nd Floor, Skipton House, 80 London Road, London SE1 6LH, UK; d School of Medicine, Pharmacy and Health, Durham University, Stockton on Tees TS17 6BH, UK; e Cambridge Centre for Health Services Research, University of Cambridge, Cambridge CB2 0SR, UK

**Keywords:** Breast neoplasms, Early detection of cancer, Signs and symptoms, Primary health care, Female, Delayed diagnosis, Early diagnosis

## Abstract

**Introduction:**

Most symptomatic women with breast cancer have relatively short diagnostic intervals but a substantial minority experience prolonged journeys to diagnosis. Atypical presentations (with symptoms other than breast lump) may be responsible.

**Methods:**

We examined the presenting symptoms of breast cancer in women using data from a national audit initiative (n = 2316). Symptoms were categorised topographically. We investigated variation in the length of the patient interval (time from symptom onset to presentation) and the primary care interval (time from presentation to specialist referral) across symptom groups using descriptive analyses and quantile regression.

**Results:**

A total of 56 presenting symptoms were described: breast lump was the most frequent (83%) followed by non-lump breast symptoms, (e.g. nipple abnormalities (7%) and breast pain (6%)); and non-breast symptoms (e.g. back pain (1%) and weight loss (0.3%)).

Greater proportions of women with ‘non-lump only’ and ‘both lump and non-lump’ symptoms waited 90 days or longer before seeking help compared to those with ‘breast lump only’ (15% and 20% vs. 7% respectively). Quantile regression indicated that the differences in the patient interval persisted after adjusting for age and ethnicity, but there was little variation in primary care interval for the majority of women.

**Conclusions:**

About 1 in 6 women with breast cancer present with a large spectrum of symptoms other than breast lump. Women who present with non-lump breast symptoms tend to delay seeking help. Further emphasis of breast symptoms other than breast lump in symptom awareness campaigns is warranted.

## Highlights

• A minority of women with breast cancer experience substantial diagnostic delays.
• Our findings suggest that around 1 in 6 women had symptoms other than breast lump.
• On average, women experienced longer patient intervals than primary care intervals.
• Women with ‘non-lump’ or ‘both lump and non-lump’ symptoms delayed seeking help.
• Symptom awareness campaigns should further emphasise non-lump breast symptoms.

## 1 Introduction

Breast lump is the most common presenting symptom among women with breast cancer and has relatively high predictive value for malignancy [1], [2]. Consequently, it has long been the focus of public health education campaigns about cancer symptom awareness [3], [4]. Although women with breast cancer typically experience short diagnostic intervals compared to other cancer patients, some women continue to experience long diagnostic intervals [2], [5], [6], [7], [8]. This is concerning as longer intervals to diagnosis have been shown to be associated with lower five-year survival of breast cancer patients, and additionally, a prolonged diagnostic experience may lead to poorer experience of subsequent cancer care [9], [10], [11]. Further, inequalities in stage at diagnosis and survival of breast cancer patients have been linked to variation in the length of the patient interval [12], [13], [14].

Prior literature exploring reasons for delayed help-seeking suggests that women subsequently diagnosed with breast cancer may attribute non-lump breast symptoms to other non-malignant causes such as hormonal changes, trauma, or breastfeeding [15], [16], [17]. While this provides an explanation of why some women may experience long intervals to presentation, there has been limited examination of diagnostic timeliness using population-based studies and large representative samples of women with breast cancer. Moreover, existing studies often dichotomise presenting symptoms based on the presence or absence of breast lump, limiting the appreciation of the large spectrum of presenting symptoms within the ‘non-lump’ breast symptoms category [18], [19], [20], [21].

Motivated by the above considerations, we aimed to describe the diverse range of presenting symptoms in a large representative sample of women with breast cancer in England, and to examine associations between different symptomatic presentations and the length of diagnostic intervals. Our broader aim was to provide underpinning evidence to inform the content and targeting of public health campaigns and decision-support interventions in primary care.

## 2 Materials and methods

### 2.1 Data

We analysed data from the English National Audit of Cancer Diagnosis in Primary Care (2009–10) which collected information on the diagnostic pathway of cancer patients in 14% of all English general practices [22]. Patients were selected on a continuous basis, minimising the potential for selection bias. The patient population was representative of the age, sex, and cancer case-mix of incident cancer patients in England, and participating practices were also comparable to non-participating practices in respective (former) Cancer Networks [22], [23]. Our analysis sample comprised 2316 women with breast cancer with complete and valid information on age, ethnicity, and presenting symptoms. Among these women, 1883 (81%), 2201 (95%), and 2002 (86%) had complete information on the patient interval, the primary care interval, and the number of pre-referral consultations respectively (Supplementary Fig. A.1). Women with missing interval or pre-referral consultation data were less likely to have presented in general practice, or were older (70 years or over) without evidence for variation by ethnicity, symptom group, or number of symptoms (data not shown).

### 2.2 Presenting symptoms

As part of the audit, general practitioners within participating practices provided free-text information on the main presenting symptom(s) of patients, based on information in their records. Informed by the principles of natural language processing (NLP), free-text descriptions were coded into symptoms without using any prior construct definitions or restrictions [24]. Symptom were initially assigned by MMK, and subsequently verified by GL and GPR. Where there was diverging opinion, consensus was reached by discussion.

### 2.3 Diagnostic intervals

As previously reported, the length of the patient and primary care intervals were derived based on information in the patients’ primary care records [25], [26]. Concordant with international consensus statements, the patient interval was defined as the number of days between symptom onset and the first presentation, and the primary care interval as the number of days between first presentation and the first specialist referral [27]. The number of pre-referral consultations was also examined, as a strongly correlated marker of the length of the primary care interval [6]. Pre-referral consultations were parameterised as a binary outcome (1 pre-referral consultation vs 2 or more pre-referral consultations) as the great majority of women (90%) had a single consultation.

### 2.4 Analytic methods

Firstly, we described the frequency of recorded presenting symptoms and associated exact confidence intervals, and the distribution of the patient and primary care intervals for each symptom among women with complete interval values. Beyond summarising mean, median and key centile interval values, we have also reported the proportion of women with each symptom that experienced 2 or more pre-referral consultations [6]. Additionally, we calculated the proportion of women with interval values exceeding 90 days, given prior evidence of poorer survival among women experiencing diagnostic intervals of 3 months or longer [11].

We developed a taxonomy of presenting symptoms by classifying individual symptoms into three main symptom categories: (a) *breast lump*, (b) *non-lump breast symptoms* (including breast pain, breast skin or shape abnormalities and nipple abnormalities), and (c) *non-breast symptoms* (including fatigue, breathlessness, axillary symptoms, neck lump, and back pain) (see Fig. 2 and Fig. A.2 in Supplementary materials). Some women had multiple symptoms across different symptom categories. From the resulting seven combinations of the three symptom categories, we focused on the four largest groups (‘lump’, ‘lump and non-lump’, ‘non-lump’, and ‘non-breast’).

We used Kruskal-Wallis and Chi-squared tests to compare observed diagnostic intervals and the number of pre-referral consultations by symptom groups, and other covariates. Subsequently, regression was used to examine the variation in patient and primary care intervals by symptom group adjusted for age and ethnicity. Specifically, as the outcome data (length of patient interval and primary care interval) were highly right-skewed, a continuity correction and log-transformation was applied to both variables before using quantile regression across different centiles of interest, and significance testing was based on bootstrapping. Detailed methods and findings of quantile regression modelling are available in the Supplementary materials. All analyses were conducted in STATA SE v.13 (StataCorp, College Station, TX, USA).

## 3 Results

### 3.1 Symptom signature of breast cancer – individual symptoms

A total of 2316/2783 (83%) of symptomatic women with breast cancer were included in the analysis (see Supplementary Fig. A.1 for sample derivation). Among them, 2543 symptoms were recorded, averaging 1.1 symptoms per woman. A total of 56 distinct presenting symptoms were reported in the study population (Table 1), in 95 unique phenotypes. Breast lump was the most common symptom, recorded in about four-fifths of all women (83%). The next most commonly reported presenting symptoms were nipple abnormalities (7%), breast pain (6%), and breast skin abnormalities (2%).

**Table 1:** Frequencies of the 23 most common symptoms (with a relative frequency of 0.2% or more) among 2316 women with breast cancer included in analysis; see Table A.1 in the Supplementary material for full list of 56 symptoms.

Symptom	Symptom signature and frequency		Pre-presentation		Post-presentation		
	No of women	% Relative frequency (95% CI)	Patient Interval Median (IQR) 90th^b^ (n = 1883)	% Patient Interval >90 days^b^ (95% CI)	Primary Care Interval Median (IQR) 90th^b^ (n = 2201)	% Primary Care Interval >90 days^b^ (95% CI)	% 2+ pre-referral consultations^b^ (n = 2002)
Breast lump	1922	83.0% (81.4–84.5%)	7 (1–27) 75	8% (7–9%)	0 (0–0) 3	1% (1–2%)	6%
Nipple abnormalities	158	6.8% (5.9–7.9%)	17 (2–71) 275	23% (17–31%)	0 (0–1) 7	1% (0.4–5%)	12%
Breast pain	149	6.4% (5.5–7.5%)	10 (3–41) 96	12% (8–19%)	0 (0–3) 34	3% (1–7%)	20%
Breast skin abnormalities	46	2.0% (1.5–2.6%)	13 (1–30) 129	10% (4–24%)	0 (0–1) 3	2% (0.4–12%)	8%
Axillary lump	27	1.2% (0.8–1.7%)	2.5 (0–12) 15	0% (0–15%)	0 (0–14) 34	4% (1–18%)	36%
Breast ulceration	25	1.1% (0.7–1.6%)	122 (0–276) 594	56% (27–81%)	0 (0–1) 1	0% (0–15%)	7%
Back pain	24	1.0% (0.7–1.5%)	9.5 (1–51) 107.5	10% (3–30%)	21 (0–105) 145	26% (13–46%)	65%
Breast contour abnormalities	17	0.7% (0.5–1.2%)	5 (4–18) 184	15% (4–42%)	0 (0–1) 3	0% (0–20%)	7%
Breast infection or inflammation	15	0.6% (0.4–1.1%)	2.5 (0–30) 366	21% (8–48%)	9 (0–23) 37	7% (1–31%)	60%
Breast swelling	14	0.6% (0.4–1.0%)	3.5 (0–14)^a^	10% (2–40%)	0 (0–3.5) 8	0% (0–24%)	15%
Musculoskeletal pain	14	0.6% (0.4–1.0%)	0.5 (0–22)^a^	10% (2–40%)	54 (0–187.5) 399	25% (9–53%)	75%
Breathlessness	11	0.5% (0.3–0.8%)	5 (0–35.5)^a^	0% (0–49%)	1 (0–10.5)^a^	0% (0–32%)	57%
Breast rash	10	0.4% (0.2–0.8%)	0 (0–16)^a^	0% (0–39%)	0 (0–7)^a^	0% (0–32%)	20%
Neck lump or lymph node abnormalities	9	0.4% (0.2–0.7%)	0 (0–10)^a^	0% (0–39%)	4.5 (0–19.5)^a^	0% (0–32%)	29%
Abdominal pain	8	0.3% (0.2–0.7%)	39 (18–62)^a^	17% (3–56%)	3 (2–6)^a^	0% (0–43%)	71%
Other breast abnormalities	8	0.3% (0.2–0.7%)	6 (0–8)^a^	0% (0–43%)	0 (0–98)^a^	33% (10–70%)	14%
Chest pain	8	0.3% (0.2–0.7%)	18 (10–43)^a^	0% (0–32%)	24 (9.5–83)^a^	25% (7–59%)	75%
Fatigue or weakness	7	0.3% (0.1–0.6%)	10.5 (1.5–33)^a^	0% (0–49%)	2 (0–27)^a^	14% (3–51%)	29%
Weight Loss	6	0.3% (0.1–0.6%)	56 (51–61)^a^	0% (0–66%)	18 (11–22)^a^	0% (0–43%)	60%
Cough	6	0.3% (0.1–0.6%)	5.5 (0–11)^a^	0% (0–66%)	13.5 (6.5–38)^a^	0% (0–49%)	60%
Axillary pain	5	0.2% (0.1–0.5%)	15 (0–126)^a^	33% (6–79%)	5 (1–8)^a^	0% (0–43%)	40%
Breast bruising	5	0.2% (0.1–0.5%)	7 (7–14)^a^	0% (0–43%)	0 (0–8)^a^	0% (0–43%)	40%
Oedema of upper limb	5	0.2% (0.1–0.5%)	76 (19–133)^a^	50% (10–91%)	0.5 (0–1)^a^	0% (0–49%)	0%
Total	2316	–	7 (1–28) 80	9% (8–10%)	0 (0–1) 7	2% (1–2%)	10%

NB Symptom frequencies do not add up to 100% as some women had more than one symptom.

a 90th centile PI and PCI values not shown for symptoms where there were <10 patients with non-missing values.

b 19%, 5%, and 14% of all observations had missing information on the patient interval, the primary care interval, and the number of pre-referral consultations respectively. For exact proportion by symptom please see Table A.1 in the Supplementary material.

Overall, 164 women (9% of those with patient interval values) waited longer than 90 days before seeking help. Among the larger non-lump breast symptoms, more than one in five women with breast ulceration (50%), nipple abnormalities (23%) and breast infection or inflammation (21%) had patient intervals of more than 90 days (Table 1). In contrast to the substantial proportion of women with patient intervals longer than 3 months (9%, as above), only 2% of women had recorded primary care interval values of 90 days or longer. This small group of women tended to have symptoms such as non-specific breast abnormalities, back pain, musculoskeletal pain, chest pain, and fatigue or weakness.

### 3.2 Variation in diagnostic intervals by major symptom group

**Fig. 1:**
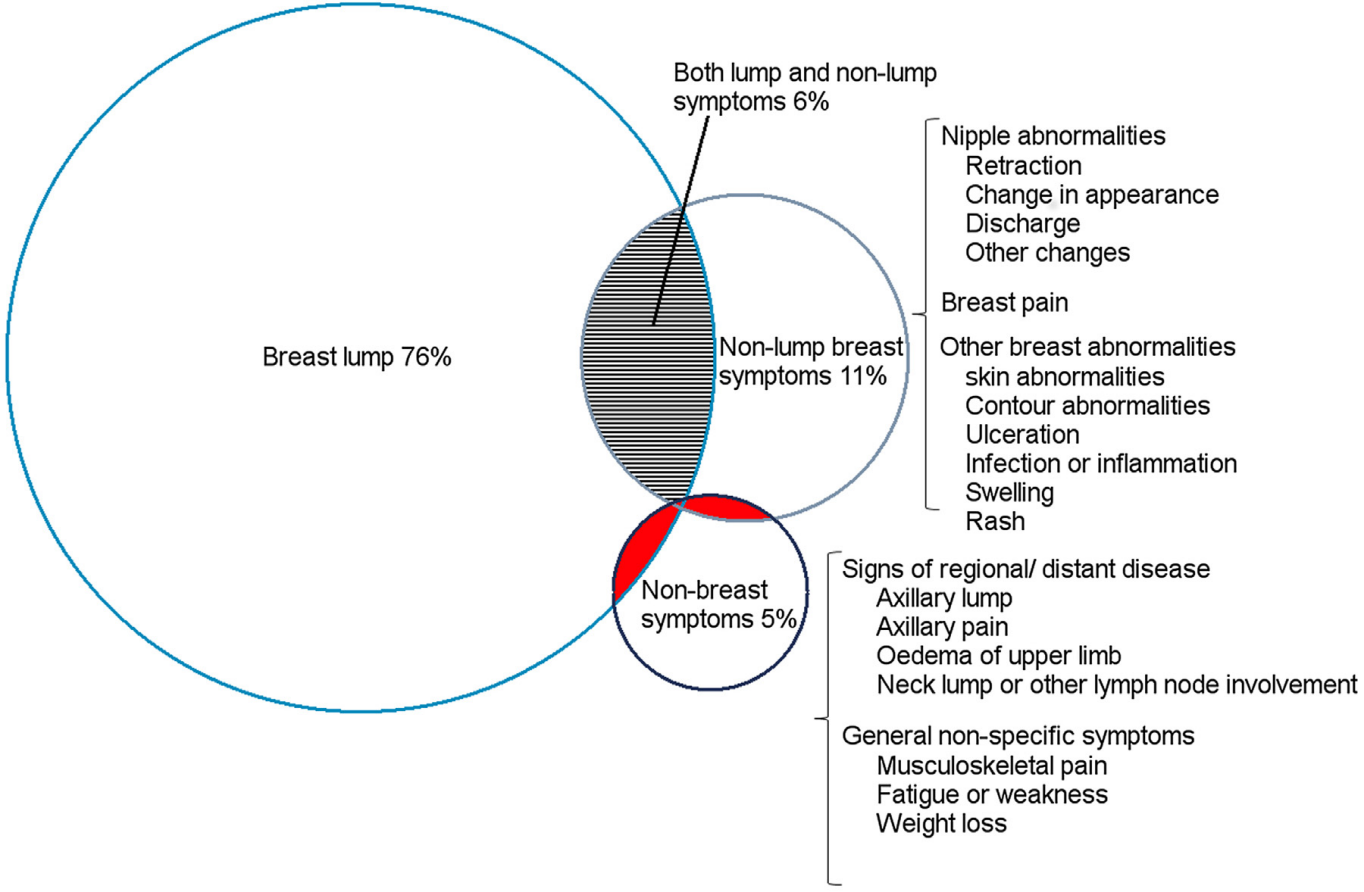
Venn diagram depicting the four largest symptom groups in 2316 breast cancer patients. The three shaded groups in red were not investigated due to small numbers: breast lump and non-breast symptoms (n = 12), non-lump breast symptoms and non-breast symptoms (n = 7), and breast lump, non-lump breast symptoms, and non-breast symptoms (n = 1). The full symptom taxonomy is presented in Fig. A.2. in the Supplementary material (For interpretation of the references to colour in this figure legend, the reader is referred to the web version of this article.)

The vast majority (99%) of women belonged to one of four symptom groups: ‘lump only’ (76%); ‘non-lump only’ (11%); ‘both lump and non-lump’ (6%); and ‘non-breast symptoms’ (5%) (Fig. 1). There was no difference in frequency of symptom groups by age group of ethnicity (Supplementary Table A.2).

**Fig. 2:**
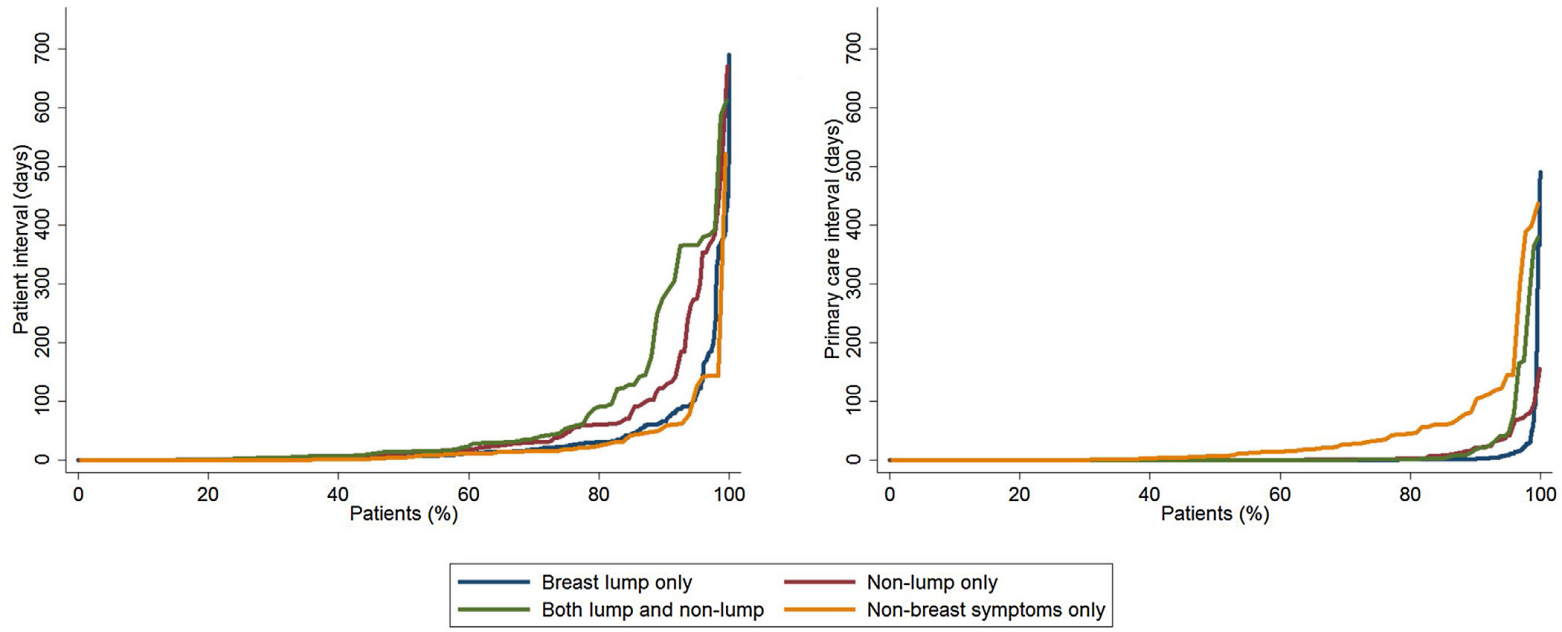
Quantile plot distribution of the patient (left) and primary care (right) intervals by symptom group. Note that curves tend to separate towards the upper end of the distribution. Data relate to the four largest presenting symptom groups (see main text). (Please refer to the web version of this article for a colour version.)

As most of the variation in interval length between different symptom groups was concentrated at the long right tail of the distribution, we hereafter describe the 90th centile values in addition to the median value. Overall, the patient interval was substantially longer than the primary care interval (median 7 vs 0 days, and 90th centile 80 vs 7 days, respectively; Table 2 and Fig. 2).

**Table 2:** Descriptive statistics of the patient interval (n = 1878^a^ and primary care interval (n = 2194^a^) in symptomatic women with breast cancer. Quantile regression modelling output is presented in the Supplementary material.

Symptom group	Median (IQR) 90th	P-value	% women >90 days (95% CI)
Patient Interval^a^			
All women	7 (1–28) 80	–	9% (8–10%)
Breast lump only	7 (1–24) 66	<**0.001**^b^	7% (6–9%)
Non-lump only	12 (2–46) 126		15% (11–20%)
Lump and non-lump	14 (3–54) 276		20% (14–29%)
Non-breast symptoms	4 (0–18) 59		6% (2–12%)
White	7 (1–28) 80	0.509^c^	9% (8–10%)
Non-white	6 (0–30) 78		8% (5–14%)
<50 years	7 (1–27) 66	0.148^c^	7% (5–10%)
50–69 years	7 (1–25) 72		8% (6–10%)
70+ years	7 (1–31) 92		11% (9–13%)

Primary Care Interval^a^			
All women	0 (0–1) 7	–	2% (1–2%)
Breast lump only	0 (0–0) 2	**<0.001**^b^	1% (1–2%)
Non-lump only	0 (0–1) 21		1% (0.4–4%)
Lump and non-lump	0 (0–1) 18		4% (2–8%)
Non-breast symptoms	7 (0–34) 105		10% (6–17%)
White	0 (0–1) 7	0.620^c^	2% (1–2%)
Non-white	0 (0–0) 10		1% (0.3–5%)
<50 years	0 (0–1) 15	**0.016**^c^	3% (2–5%)
50–69 years	0 (0–0) 4		1% (1–2%)
70+ years	0 (0–1) 3		1% (1–2%)

Bold denotes p < 0.05.

a K19% and 5% of women had missing information on the patient interval and the primary care interval respectively.

b Kruskal-Wallis tests.

c Chi-squared tests.

#### 3.2.1 Patient interval

There was strong evidence for variation in the patient interval by symptom group (p < 0.001). Women with ‘lump only’ symptoms had median (90th centile) patient interval values of 7 (66) days. In contrast, those with ‘non-lump only’ or ‘both lump and non-lump’ symptoms had median (90th centile) intervals of 12 (126) days and 14 (276) days, respectively, while women with ‘non-breast symptoms’ had shorter intervals (of 4 (59) days) (Table 2). Observed patterns of variation in the patient interval by symptom group remained largely unchanged after adjusting for age group and ethnicity. There was no evidence for variation in the length of the patient interval by age or ethnicity at any of the quantile points examined (Supplementary Table A.4).

#### 3.2.2 Primary care interval

Observed primary care interval values also varied by symptom group: women presenting with ‘lump only’ had the shortest median (90th centile) intervals (0 (2) days), while those with ‘non-breast’ symptoms had the longest intervals (7 (105) days), respectively (Table 2). Concordant patterns of variation by symptom group were apparent when examining the proportion of women with 2 or more pre-referral consultations (Supplementary Table A.3). Adjusting for differences in age group and ethnicity, symptom groups other than the ‘lump only’ group had longer intervals to referral, but these differences were only significant in the upper centiles (Supplementary Table A.4 and Fig. 2).

## 4 Discussion

About 1 in 6 women with breast cancer presented without a breast lump, instead experiencing a wide spectrum of symptoms before seeking help. The length of the patient and the primary care intervals varied by symptom group, particularly in the upper centiles of the distribution. Women in the ‘non-lump only’ and ‘both lump and non-lump’ symptom groups had longer median patient intervals compared to those with ‘breast lump only’. Similar associations were seen post-presentation, although on average women had appreciably shorter primary care intervals than patient intervals.

To our knowledge, this is the first and largest study to examine associations between a range of presenting symptoms of breast cancer and the length of the patient and the primary care intervals. The present analysis substantially amplifies previous findings in this field, providing evidence of notable differences in diagnostic timeliness by the symptoms of breast cancer [9], [16]. Regarding the symptom signature of breast cancer, a previous study using Read-coded electronic primary care data reported similar proportions of non-lump breast symptoms to those observed in our study [2], but we have been able to describe a wide range of presenting symptoms in substantially greater detail than the categorisations used to date.

The study setting is within a publicly funded health system where patients have free access to primary care services and primary care physicians act as gate-keepers to specialist services. We would not expect health system factors to affect the process of symptom appraisal by women, but patient intervals may be longer in healthcare systems without universal healthcare coverage. In contrast, although in theory gate-keeping may be associated with prolonged primary care intervals, in practice we observed very short primary care intervals for the majority of women in our study [28]. Therefore we do not believe that the context of our study substantially affects the relevance of the findings, particularly in relation to the patient interval which was the dominant source of delay.

There are several limitations that should be acknowledged. The validity and completeness of symptom information is dependent on patients accurately recalling and describing their symptoms during the consultation, and on doctors accurately interpreting and recording them. Additionally, as patient records were examined retrospectively (and in the knowledge of the patient’s diagnosis), non-specific, particularly non-breast, symptoms may have been under-captured by the audit. There were missing outcome data regarding intervals and number of consultations for a minority of women, in proportions comparable to previous studies in this field [7], [29], [30], [31]. Women who did not first present in primary care and were older were more likely to have missing data but were otherwise similar across other characteristics of interest. We were unable to examine variation in diagnostic intervals by level of deprivation or other patient-level characteristics such as health literacy or history of screening participation as this information was not captured by the audit, although the length of patient intervals by symptom may vary by socio-economic status [12], [32]. Although we were able to describe the overall symptom signature of breast cancer in appreciable detail, associations with diagnostic timeliness measures were analysed using aggregate symptom groups due to sample size limitations regarding rarer individual symptoms, particularly non-breast symptoms. Lastly, while data relate to a recent annual period, further monitoring of associations between symptoms and diagnostic intervals in more recent cohorts will be useful.

The present study provides detailed evidence about the symptom signature of breast cancer, and the frequencies and diagnostic intervals associated with different symptoms, which could inform the design of public health campaigns. Existing examples of population- or person-level breast awareness interventions that encompass both lump and non-lump symptoms of the breast include the English breast “Be Clear on Cancer” campaign and the “Promoting Early Presentation” intervention [33], [34], [35]. Our findings support a continued shift in emphasis of awareness interventions to encompass the likely importance of ‘non-lump’ breast symptoms.

Beyond considering the symptom signature and associated diagnostic intervals, the design of awareness campaigns should also reflect the predictive value of symptoms for a given malignancy. Currently, there is little relevant evidence beyond that for breast lump, but some non-lump breast symptoms (such as nipple eczema or breast ulceration) may have equal or greater positive predictive values for breast cancer [36], [37].

Women in the ‘both lump and non-lump’ group had longer patient intervals compared to those with ‘breast lump only’ group. This is somewhat puzzling given that breast lump, which is associated with shorter intervals, is present in both groups. This may reflect a higher tendency for women normalise a lump in the breast in the presence of other non-lump breast symptoms [12]. Relatedly, previous research indicates that among women with prolonged patient intervals (12 weeks or longer), some had initially experienced non-lump breast symptoms and then had subsequently developed a lump by the time of (delayed) presentation [18]. Prospective designs such as those employed by the SYMPTOM studies in England may help explore the time sequence of symptom occurrence and diagnostic intervals, although logistical constraints may limit sample size and power [30].

The majority of women had much shorter intervals post-presentation than pre-presentation (1 in 2 women with breast cancer in our study had a primary care interval of 0 days) and there was no evidence for variation in the median primary care interval by symptom group. The small minority of women who presented with ‘non-breast symptoms’ (e.g. back pain or breathlessness) however had substantially longer primary care intervals compared to those with breast lump or non-lump breast symptoms. Shortening diagnostic intervals in such women will improve patient experience, but may not lead to better clinical outcomes given that distant symptoms might represent late stage disease [10]. Identifying these women is also likely to be challenging, due to the low predictive values of these symptoms for breast cancer. New diagnostic services for non-specific symptoms such as the ‵Danish three-legged strategy’ and those being piloted by the those being piloted by Accelerate, Coordinate, Evaluate (ACE) initiative in England may be of particular value in this regard [38], [39].

## 5 Conclusions

In conclusion, this study provides a detailed description of the symptom signature at presentation among women subsequently diagnosed with breast cancer, and confirms an association between non-lump presenting symptoms of the breast and prolonged diagnostic intervals. Our findings highlight the need for healthcare interventions to support the diagnostic process in women with atypical presentations; and support efforts to focus on non-lump breast symptoms through public health education campaigns in order to facilitate earlier presentation.

## Conflict of interest

None.

## Funding

This work was supported by a grant from the UK Department of Health (no. 106/0001). This work was part of the programme of the Policy Research Unit in Cancer Awareness, Screening and Early Diagnosis. The Policy Research Unit in Cancer Awareness, Screening, and Early Diagnosis receives funding for a research programme from the Department of Health Policy Research Programme. It is a collaboration between researchers from seven institutions (Queen Mary University of London, University College London, King’s College London, London School of Hygiene and Tropical Medicine, Hull York Medical School, Durham University and Peninsula Medical School/University of Exeter). GL is supported by Cancer Research UK Clinician Advanced Scientist Fellowship A18180.

## Authorship contribution

MMK, GPR, and GL conceived the study. Data acquisition and quality control was done by MMK and SMc. MMK conducted all statistical analyses with assistance from GAA and SMc. MMK wrote the first draft of the manuscript, and prepared the tables and figures, supervised by GL. All authors substantially contributed to the interpretation of the results, revised the manuscript and approved the final version of the manuscript.

## References

[1] Walker S., Hyde C., Hamilton W. (2014). Risk of breast cancer in symptomatic women in primary care: a case-control study using electronic records. Br. J. Gen. Pract..

[2] Redaniel M.T., Martin R.M., Ridd M.J., Wade J., Jeffreys M. (2015). Diagnostic intervals and its association with breast, prostate, lung and colorectal cancer survival in England: historical cohort study using the clinical practice research datalink. PLoS One.

[3] Janz N.K., Becker M.H., Anderson L.A., Marcoux B.C. (1989). Interventions to enhance breast self-examination practice: a review. Publ. Health Rev..

[4] Roberts M.M., French K., Duffy J. (1984). Breast cancer and breast self-examination: what do Scottish women know?. Soc. Sci. Med..

[5] Baughan P., O’Neill B., Fletcher E. (2009). Auditing the diagnosis of cancer in primary care: the experience in Scotland. Br. J. Cancer.

[6] Lyratzopoulos G., Abel G.A., McPhail S., Neal R.D., Rubin G.P. (2013). Measures of promptness of cancer diagnosis in primary care: secondary analysis of national audit data on patients with 18 common and rarer cancers. Br. J. Cancer.

[7] Hansen R.P., Vedsted P., Sokolowski I., Søndergaard J., Olesen F. (2011). Time intervals from first symptom to treatment of cancer: a cohort study of 2,212 newly diagnosed cancer patients. BMC Health Serv. Res..

[8] Neal R.D., Din N.U., Hamilton W., Ukoumunne O.C., Carter B., Stapley S., Rubin G. (2014). Comparison of cancer diagnostic intervals before and after implementation of NICE guidelines: analysis of data from the UK General Practice Research Database. Br. J. Cancer.

[9] Webber C., Jiang L., Grunfeld E., Groome P.A. (2017). Identifying predictors of delayed diagnoses in symptomatic breast cancer: a scoping review. Eur. J. Cancer.

[10] Mendonca S.C., Abel G., Saunders C.L., Wardle J., Lyratzopoulos G. (2016). Pre-referral general practitioner consultations and subsequent experience of cancer care: evidence from the English Cancer Patient Experience Survey. Eur. J. Cancer Care (Engl.).

[11] Richards M.A., Westcombe A.M., Love S.B., Littlejohns P., Ramirez A.J. (1999). Influence of delay on survival in patients with breast cancer: a systematic review. Lancet.

[12] Marcu A., Lyratzopoulos G., Black G., Vedsted P., Whitaker K.L. (2016). Educational differences in likelihood of attributing breast symptoms to cancer: a vignette-based study. Psychooncology.

[13] Lyratzopoulos G., Abel G. (2013). Earlier diagnosis of breast cancer: focusing on symptomatic women. Nat. Rev. Clin. Oncol..

[14] Rutherford M.J., Hinchliffe S.R., Abel G.A., Lyratzopoulos G., Lambert P.C., Greenberg D.C. (2013). How much of the deprivation gap in cancer survival can be explained by variation in stage at diagnosis: an example from breast cancer in the East of England. Int. J. Cancer.

[15] O’Mahony M., McCarthy G., Corcoran P., Hegarty J. (2013). Shedding light on women’s help seeking behaviour for self discovered breast symptoms. Eur. J. Oncol. Nurs..

[16] Ramirez A.J., Westcombe A.M., Burgess C.C., Sutton S., Littlejohns P., Richards M.A. (1999). Factors predicting delayed presentation of symptomatic breast cancer: a systematic review. Lancet.

[17] Khakbazan Z., Taghipour A., Roudsari R.L., Mohammadi E. (2014). Help seeking behavior of women with self-discovered breast cancer symptoms: a meta-ethnographic synthesis of patient delay. PLoS One.

[18] Burgess C., Ramirez A., Richards M., Love S. (1998). Who and what influences delayed presentation in breast cancer?. Br. J. Cancer.

[19] Redondo M., Rodrigo I., Pereda T., Funez R., Acebal M., Perea-Milla E., Jimenez E. (2009). Prognostic implications of emergency admission and delays in patients with breast cancer. Support. Care Cancer.

[20] Poum A., Promthet S., Duffy S.W., Parkin D.M. (2014). Factors associated with delayed diagnosis of breast cancer in northeast Thailand. J. Epidemiol..

[21] Innos K., Padrik P., Valvere V., Eelma E., Kütner R., Lehtsaar J., Tekkel M. (2013). Identifying women at risk for delayed presentation of breast cancer: a cross-sectional study in Estonia. BMC Publ. Health.

[22] Rubin G.P., McPhail S., Elliot K., McPhail S. (2011). Royal College of General Practitioners.

[23] Lyratzopoulos G., Abel G.A., McPhail S., Neal R.D., Rubin G.P. (2013). Gender inequalities in the promptness of diagnosis of bladder and renal cancer after symptomatic presentation: evidence from secondary analysis of an English primary care audit survey. BMJ Open.

[24] Doan S., Conway M., Phuong T.M., Ohno-Machado L. (2014). Natural language processing in biomedicine: a unified system architecture overview. Methods Mol. Biol..

[25] Keeble S., Abel G.A., Saunders C.L., McPhail S., Walter F.M., Neal R.D., Rubin G.P., Lyratzopoulos G. (2014). Variation in promptness of presentation among 10,297 patients subsequently diagnosed with one of 18 cancers: evidence from a National Audit of Cancer Diagnosis in Primary Care. Int. J. Cancer.

[26] Lyratzopoulos G., Saunders C.L., Abel G.A., McPhail S., Neal R.D., Wardle J., Rubin G.P. (2015). The relative length of the patient and the primary care interval in patients with 28 common and rarer cancers. Br. J. Cancer.

[27] Weller D., Vedsted P., Rubin G., Walter F.M., Emery J., Scott S., Campbell C., Andersen R.S., Hamilton W., Olesen F., Rose P., Nafees S., van Rijswijk E., Hiom S., Muth C., Beyer M., Neal R.D. (2012). The Aarhus statement: improving design and reporting of studies on early cancer diagnosis. Br. J. Cancer.

[28] Vedsted P., Olesen F. (2011). Are the serious problems in cancer survival partly rooted in gatekeeper principles? An ecologic study. Br. J. Gen. Pract..

[29] Leiva A., Esteva M., Llobera J., Macià F., Pita-Fernández S., González-Luján L., Sánchez-Calavera M.A., Ramos M. (2017). Time to diagnosis and stage of symptomatic colorectal cancer determined by three different sources of information: a population based retrospective study. Cancer Epidemiol..

[30] Walter F.M., Emery J.D., Mendonca S., Hall N., Morris H.C., Mills K., Dobson C., Bankhead C., Johnson M., Abel G.A., Rutter M.D., Hamilton W., Rubin G.P. (2016). Symptoms and patient factors associated with longer time to diagnosis for colorectal cancer: results from a prospective cohort study. Br. J. Cancer.

[31] Walter F.M., Mills K., Mendonça S.C., Abel G.A., Basu B., Carroll N., Ballard S., Lancaster J., Hamilton W., Rubin G.P., Emery J.D. (2016). Symptoms and patient factors associated with diagnostic intervals for pancreatic cancer (SYMPTOM pancreatic study): a prospective cohort study. Lancet Gastroenterol. Hepatol..

[32] Marcu A., Black G., Vedsted P., Lyratzopoulos G., Whitaker K.L. (2017). Educational differences in responses to breast cancer symptoms: a qualitative comparative study. Br. J. Health Psychol..

[33] Public Health England, Be Clear on Cancer (2016). http://www.nhs.uk/be-clear-on-cancer/.

[34] Kaushal A., Ramirez A.J., Warburton F., Forster A.S., Linsell L., Burgess C., Tucker L., Omar L., Forbes L.J. (2016). Promoting early presentation intervention sustains increased breast cancer awareness in older women for three years: a randomized controlled trial. J. Med. Screen..

[35] Campbell J., Pyer M., Rogers S., Jones J., Ramirez A.J., Forbes L.J.L. (2016). Promoting early presentation of breast cancer in women over 70 years old in general practice. J. Publ. Health (Bangk.).

[36] Huggenberger I.K., Andersen J.S. (2015). Predictive value of the official cancer alarm symptoms in general practice-a systematic review. Dan. Med. J..

[37] Dalberg K., Hellborg H., Wärnberg F. (2008). Paget’s disease of the nipple in a population based cohort. Breast Cancer Res. Treat..

[38] Fuller E., Fitzgerald K., Hiom S. (2016). Accelerate, coordinate, evaluate programme: a new approach to cancer diagnosis. Br. J. Gen. Pract..

[39] Vedsted P., Olesen F. (2015). A differentiated approach to referrals from general practice to support early cancer diagnosis—the Danish three-legged strategy. Br. J. Cancer.

