# Supplementary material for: Typical and atypical presenting symptoms of breast cancer and their associations with diagnostic intervals: Evidence from a national audit of cancer diagnosis
Source: Cancer Epidemiol. 2017 Jun;48:140–6. doi: 10.1016/j.canep.2017.04.010 (PMC5482318; doi:10.1016/j.canep.2017.04.010)
Supplement: Supplementary file 1 [file mmc1.docx]

# Appendix A

## Figure A.1 Flow chart of sample derivation

2,882 patients with symptomatic breast cancer in the NACDPC 2009/10

2,779 patients

Excluded 103 (3.6%)

Patients with male breast cancer

Excluded 49 (1.8%)

Patients missing information on age

2,730 patients

2,411 patients

Excluded 319 (11.7%)

Patients missing information on ethnicity

2,316 patients

Excluded 95 (3.9%)

Patients missing valid symptom information

## Figure A.2 Breast symptom taxonomy

## Table A.1 Full list of symptoms among women with breast cancer (n=2,316)

|  | **Symptom signature and frequency** | | **Pre-presentation** | | | **Post-presentation** | | | | |
| --- | --- | --- | --- | --- | --- | --- | --- | --- | --- | --- |
|  | **N** | **% relative frequency**  **(95% CI)** | **Patient Interval Median (IQR) 90^th^** | **% Patient Interval > 90 days**  **(95^th^ CI)** | **% missing** | **Primary Care Interval Median (IQR) 90^th^** | **% Primary Care Interval > 90 days**  **(95^th^ CI)** | **% missing** | **% 2+ pre-referral consultations** | **% missing** |
| Breast lump | 1922 | 83.0% (81.4-84.5%) | 7 (1 - 27) 75 | 8% (7-9%) | 18% | 0 (0 - 0) 3 | 1% (1-2%) | 4% | 6% | 13% |
| Nipple abnormalities | 158 | 6.8% (5.9-7.9%) | 17 (2 - 71) 275 | 23% (17-31%) | 21% | 0 (0 - 1) 7 | 1% (0.4-5%) | 3% | 12% | 15% |
| Breast pain | 149 | 6.4% (5.5-7.5%) | 10 (3 - 41) 96 | 12% (8-19%) | 12% | 0 (0 - 3) 34 | 3% (1-7%) | 3% | 20% | 8% |
| Breast skin abnormalities | 46 | 2.0% (1.5-2.6%) | 13 (1 - 30) 129 | 10% (4-24%) | 15% | 0 (0 - 1) 3 | 2% (0.4-12%) | 2% | 8% | 17% |
| Axillary lump | 27 | 1.2% (0.8-1.7%) | 2.5 (0 - 12) 15 | 0% (0-15%) | 19% | 0 (0 - 14) 34 | 4% (1-18%) | 0% | 36% | 19% |
| Breast ulceration | 25 | 1.1% (0.7-1.6%) | 122 (0 - 276) 594 | 56% (27-81%) | 64% | 0 (0 - 1) 1 | 0% (0-15%) | 16% | 7% | 40% |
| Back pain | 24 | 1.0% (0.7-1.5%) | 9.5 (1 - 51) 107.5 | 10% (3-30%) | 17% | 21 (0 - 105) 145 | 26% (13-46%) | 4% | 65% | 4% |
| Breast contour abnormalities | 17 | 0.7% (0.5-1.2%) | 5 (4 - 18) 184 | 15% (4-42%) | 24% | 0 (0 - 1) 3 | 0% (0-20%) | 12% | 7% | 18% |
| Breast infection or inflammation | 15 | 0.6% (0.4-1.1%) | 2.5 (0 - 30) 366 | 21% (8-48%) | 7% | 9 (0 - 23) 37 | 7% (1-31%) | 7% | 60% | 0% |
| Breast swelling | 14 | 0.6% (0.4-1.0%) | 3.5 (0 - 14) † | 10% (2-40%) | 29% | 0 (0 - 3.5) 8 | 0% (0-24%) | 14% | 15% | 7% |
| Musculoskeletal pain | 14 | 0.6% (0.4-1.0%) | 0.5 (0 - 22) † | 10% (2-40%) | 29% | 54 (0 - 187.5) 399 | 25% (9-53%) | 14% | 75% | 14% |
| Breathlessness | 11 | 0.5% (0.3-0.8%) | 5 (0 - 35.5) † | 0% (0-49%) | 64% | 1 (0 - 10.5) † | 0% (0-32%) | 27% | 57% | 36% |
| Breast rash | 10 | 0.4% (0.2-0.8%) | 0 (0 - 16) † | 0% (0-39%) | 40% | 0 (0 - 7) † | 0% (0-32%) | 20% | 20% | 0% |
| Neck lump or lymph node abnormalities | 9 | 0.4% (0.2-0.7%) | 0 (0 - 10) † | 0% (0-39%) | 33% | 4.5 (0 - 19.5) † | 0% (0-32%) | 11% | 29% | 22% |
| Abdominal pain | 8 | 0.3% (0.2-0.7%) | 39 (18 - 62) † | 17% (3-56%) | 25% | 3 (2 - 6) † | 0% (0-43%) | 38% | 71% | 13% |
| Other breast abnormalities | 8 | 0.3% (0.2-0.7%) | 6 (0 - 8) † | 0% (0-43%) | 38% | 0 (0 - 98) † | 33% (10-70%) | 25% | 14% | 13% |
| Chest pain | 8 | 0.3% (0.2-0.7%) | 18 (10 - 43) † | 0% (0-32%) | 0% | 24 (9.5 - 83) † | 25% (7-59%) | 0% | 75% | 0% |
| Fatigue or weakness | 7 | 0.3% (0.1-0.6%) | 10.5 (1.5 - 33) † | 0% (0-49%) | 43% | 2 (0 - 27) † | 14% (3-51%) | 0% | 29% | 0% |
| Weight Loss | 6 | 0.3% (0.1-0.6%) | 56 (51 - 61) † | 0% (0-66%) | 67% | 18 (11 - 22) † | 0% (0-43%) | 17% | 60% | 17% |
| Cough | 6 | 0.3% (0.1-0.6%) | 5.5 (0 - 11) † | 0% (0-66%) | 67% | 13.5 (6.5 - 38) † | 0% (0-49%) | 33% | 60% | 17% |
| Axillary pain | 5 | 0.2% (0.1-0.5%) | 15 (0 - 126) † | 33% (6-79%) | 40% | 5 (1 - 8) † | 0% (0-43%) | 0% | 40% | 0% |
| Breast bruising | 5 | 0.2% (0.1-0.5%) | 7 (7 - 14) † | 0% (0-43%) | 0% | 0 (0 - 8) † | 0% (0-43%) | 0% | 40% | 0% |
| Oedema of upper limb | 5 | 0.2% (0.1-0.5%) | 76 (19 - 133) † | 50% (9-91%) | 60% | 0.5 (0 - 1) † | 0% (0-49%) | 20% | 0% | 0% |
| Anorexia or loss of appetite | 3 | 0.1% (0.0-0.4%) | 11 (11 - 11) † | 0% (0-79%) | 67% | 39 (17 - 61) † | 0% (0-66%) | 33% | 50% | 33% |
| Mental conditions | 3 | 0.1% (0.0-0.4%) | 13 (1 - 25) † | 0% (0-66%) | 33% | 7 (7 - 7) † | 0% (0-79%) | 67% | . | 33% |
| Other | 2 | 0.1% (0.0-0.3%) | 44 (10 - 78) † | 0% (0-66%) | 0% | 5.5 (4 - 7) † | 0% (0-66%) | 0% | 50% | 0% |
| Abdominal lump/ mass | 2 | 0.1% (0.0-0.3%) | 80.5 (18 - 143) † | 50% (9-91%) | 0% | 1 (0 - 2) † | 0% (0-66%) | 0% | 50% | 0% |
| Confusion | 2 | 0.1% (0.0-0.3%) | 13 (1 - 25) † | 0% (0-66%) | 0% | 7 (7 - 7) † | 0% (0-79%) | 50% | . | 50% |
| Headache | 2 | 0.1% (0.0-0.3%) | 43 (43 - 43) † | 0% (0-79%) | 50% | 64 (64 - 64) † | 0% (0-79%) | 50% | . | 50% |
| Other lymph node abnormalities | 2 | 0.1% (0.0-0.3%) | 16 (16 - 16) † | 0% (0-79%) | 50% | 10.5 (3 - 18) † | 0% (0-66%) | 0% | . | 0% |
| Malaise | 2 | 0.1% (0.0-0.3%) | 0 (0 - 0) † | 0% (0-79%) | 50% | 49.5 (1 - 98) † | 50% (9-91%) | 0% | . | 50% |
| Vomiting | 2 | 0.1% (0.0-0.3%) | 8 (8 - 8) † | 0% (0-79%) | 50% | 16 (16 - 16) † | 0% (0-79%) | 50% | . | 50% |
| Abdominal distension | 1 | 0.04% (0.0.-0.2%) | 0 (0 - 0) † | 0% (0-79%) | 0% | 61 (61 - 61) † | 0% (0-79%) | 0% | . | 0% |
| Ascites | 1 | 0.04% (0.0.-0.2%) | 8 (8 - 8) † | 0% (0-79%) | 0% | 16 (16 - 16) † | 0% (0-79%) | 0% | . | 0% |
| Anaemia | 1 | 0.04% (0.0.-0.2%) | - | - | 100% | - | - | 100% | . | 0% |
| Chest infection | 1 | 0.04% (0.0.-0.2%) | 0 (0 - 0) † | 0% (0-79%) | 0% | 1 (1 - 1) † | 0% (0-79%) | 0% | . | 0% |
| Loss of consciousness | 1 | 0.04% (0.0.-0.2%) | 0 (0 - 0) † | 0% (0-79%) | 0% | 0 (0 - 0) † | 0% (0-79%) | 0% | . | 0% |
| Constipation | 1 | 0.04% (0.0.-0.2%) | 18 (18 - 18) † | 0% (0-79%) | 0% | 2 (2 - 2) † | 0% (0-79%) | 0% | . | 0% |
| Cardiovascular abnormalities | 1 | 0.04% (0.0.-0.2%) | - | - | 100% | 0 (0 - 0) † | 0% (0-79%) | 0% | . | 0% |
| Diarrhoea | 1 | 0.04% (0.0.-0.2%) | - | - | 100% | 0 (0 - 0) † | 0% (0-79%) | 0% | . | 0% |
| Dizziness | 1 | 0.04% (0.0.-0.2%) | - | - | 100% | 0 (0 - 0) † | 0% (0-79%) | 0% | . | 0% |
| Epigastric pain | 1 | 0.04% (0.0.-0.2%) | 3 (3 - 3) † | 0% (0-79%) | 0% | 28 (28 - 28) † | 0% (0-79%) | 0% | . | 0% |
| Falls | 1 | 0.04% (0.0.-0.2%) | - | - | 100% | 0 (0 - 0) † | 0% (0-79%) | 0% | . | 0% |
| Fractures | 1 | 0.04% (0.0.-0.2%) | 0 (0 - 0) † | 0% (0-79%) | 0% | 0 (0 - 0) † | 0% (0-79%) | 0% | . | 0% |
| Groin pain | 1 | 0.04% (0.0.-0.2%) | - | - | 100% | - | - | 100% | . | 100% |
| Haematemesis | 1 | 0.04% (0.0.-0.2%) | 0 (0 - 0) † | 0% (0-79%) | 0% | 44 (44 - 44) † | 0% (0-79%) | 0% | . | 0% |
| Hoarseness/voice related symptoms | 1 | 0.04% (0.0.-0.2%) | - | - | 100% | 21 (21 - 21) † | 0% (0-79%) | 0% | . | 0% |
| Vision related symptoms | 1 | 0.04% (0.0.-0.2%) | - | - | 100% | 0 (0 - 0) † | 0% (0-79%) | 0% | . | 0% |
| Uncategorised lumps | 1 | 0.04% (0.0.-0.2%) | 15 (15 - 15) † | 0% (0-79%) | 0% | 57 (57 - 57) † | 0% (0-79%) | 0% | . | 100% |
| Nausea | 1 | 0.04% (0.0.-0.2%) | 1 (1 - 1) † | 0% (0-79%) | 0% | - | - | 100% | . | 0% |
| Nervous system symptoms | 1 | 0.04% (0.0.-0.2%) | 0 (0 - 0) † | 0% (0-79%) | 0% | 0 (0 - 0) † | 0% (0-79%) | 0% | . | 0% |
| Post-coital bleeding | 1 | 0.04% (0.0.-0.2%) | 0 (0 - 0) † | 0% (0-79%) | 0% | 14 (14 - 14) † | 0% (0-79%) | 0% | . | 100% |
| Speech & other cognitive abnormalities | 1 | 0.04% (0.0.-0.2%) | - | - | 100% | 0 (0 - 0) † | 0% (0-79%) | 0% | . | 0% |
| Unsteadiness or impaired mobility | 1 | 0.04% (0.0.-0.2%) | - | - | 100% | 0 (0 - 0) † | 0% (0-79%) | 0% | . | 0% |
| Unilateral weakness | 1 | 0.04% (0.0.-0.2%) | 12 (12 - 12) † | 0% (0-79%) | 0% | 45 (45 - 45) † | 0% (0-79%) | 0% | . | 0% |
| Wheeze | 1 | 0.04% (0.0.-0.2%) | 0 (0 - 0) † | 0% (0-79%) | 0% | 0 (0 - 0) † | 0% (0-79%) | 0% | . | 100% |

NB Symptom frequencies do not add up to 100% or n=2,316 as some women had more than 1 symptom.

†90^th^ centile PI and PCI values not shown for symptoms where there were <10 patients with non-missing values

PI: patient interval; PCI: primary care interval.

## Table A.2 Characteristics of breast cancer patients by symptom group

|  | **Breast lump only** | **Non-lump only** | **Both lump and non-lump** | **Non-breast symptoms only** | **Breast lump and non-breast symptoms** | **Non-lump and non-breast symptoms** | **Breast lump, non-lump, and non-breast symptoms** | **Total** | **p*** |
| --- | --- | --- | --- | --- | --- | --- | --- | --- | --- |
| Total | 1,770 (76%) | 262 (11%) | 139 (6%) | 125 (5%) | 12 (0.5%) | 7 (0.3%) | 1 (0.04%) | 2,316 (100%) | - |
| Age group |  |  |  |  |  |  |  |  |  |
| <50 years | 515 (81%) | 62 (10%) | 34 (5%) | 22 (3%) | 4 (0.6%) | 0 (0%) | 0 (0%) | 637 (100%) | 0.063 |
| 50–69 years | 586 (75%) | 99 (13%) | 43 (6%) | 49 (6%) | 2 (0.3%) | 2 (0.3%) | 0 (0%) | 781 (100%) |  |
| 70+ years | 669 (75%) | 101 (11%) | 62 (7%) | 54 (6%) | 6 0.7%) | 5 (0.6%) | 1 (0.1%) | 898 (100%) |  |
| Ethnicity |  |  |  |  |  |  |  |  |  |
| Non-white | 1,640 (76%) | 248 (12%) | 129 (6%) | 115 (5%) | 11 (0.5%) | 6 (0.3%) | 1 (0.05%) | 2,150 (100%) | 0.905 |
| White | 130 (78%) | 14 (8%) | 10 (6%) | 10 (6%) | 1 (0.6%) | 1 (0.6%) | 0 (0%) | 166 (100%) |  |

*Chi-squared test

## Table A.3 Number of pre-referral consultations by symptom group, age group, and ethnicity

| **Variable** | **% 2+ pre-referral consultations** | **P*** | **N missing** | **% missing** |
| --- | --- | --- | --- | --- |
| All women | 10% | - | 314 | 14% |
| Breast lump only | 5% |  | 236 | 15% |
| Non-lump only | 17% |  | 39 | 17% |
| Lump and non-lump | 15% |  | 16 | 13% |
| Non-breast symptoms | 54% | <0.001 | 19 | 18% |
| White | 10% |  | 287 | 15% |
| Non-white | 12% | 0.434 | 27 | 19% |
| <50 years | 14% |  | 64 | 11% |
| 50-69 years | 9% |  | 99 | 15% |
| 70+ years | 7% | <0.001 | 151 | 20% |

*Chi-squared test

## Table A.4 Quantile regression output of the patient and primary care interval

### Methods

We examined the association between symptom groups and length of the patient and primary care intervals, adjusting for age (parameterised as <50 years, 50–69 years, 70+ years) and ethnicity (white, non-white) given prior evidence suggesting their likely associations with diagnostic intervals [1,2].

Quantile regression is an established method in analysing skewed data (as is the case for diagnostic interval data), allowing relationships between outcome and predictor variables to be estimated at any point (centile) of the distribution of the interval values, where the direction and magnitude of association may differ to that of the mean [3,4]. A continuity correction and log-transformation was applied to both variables before regression modelling, and significance testing was based on bootstrapping.

### Results

#### Patient interval

Compared to women who presented with ‘breast lump only’, women in the ‘non-lump only’ group had patient intervals that were 1.6-fold (p=0.05) to 2.3-fold (p=0.003) longer at different centiles, while women with ‘lump and non-lump’ symptoms had patient intervals that were 1.9-fold (p=0.01) to 3.5-fold (p=0.001) longer at different centiles (see table below).

There was no evidence for variation in time to presentation by age group or ethnicity across all examined quantiles.

| **Variable** | **Q(0.25)**  **(95% CI)** | **Q(0.50)**  **(95% CI)** | **Q(0.75)**  **(95% CI)** | **Q(0.90)**  **(95% CI)** | **Q(0.95)**  **(95% CI)** | **Joint Wald test**  **P value** |
| --- | --- | --- | --- | --- | --- | --- |
| Breast lump only (ref) | - | - | - | - | - | **<0.001** |
| Non-lump only | 1.7 (0.7-3.8) | **1.6 (1.0-2.6)** | **1.9 (1.2-2.9)** | **2.1 (1.4-3.2)** | **2.3 (1.3-4.0)** |  |
| Lump and non-lump | **2.3 (1.1-4.8)** | **1.9 (1.2-3.2)** | 1.9 (0.9-4.0) | **3.5 (1.6-7.3)** | **2.9 (1.7-4.9)** |  |
| Non-breast symptoms | **0.3 (0.2-0.5)** | 0.7 (0.3-1.7) | 0.8 (0.5-1.2) | 0.9 (0.5-1.6) | 1.0 (0.4-2.2) |  |
| White (ref) | - | - | - | - | - | 0.779 |
| Non-white | 1.0 (0.4-2.8) | 0.9 (0.5-1.5) | 1.2 (0.8-1.7) | 1.0 (0.6-1.5) | 1.0 (0.5-1.9) |  |
| <50 years | 1.0 (0.7-1.5) | 1.2 (0.9-1.5) | 1.1 (0.8-1.5) | 1.0 (0.7-1.4) | 1.0 (0.6-1.4) | 0.421 |
| 50-69 years (ref) | - | - | - | - | - |  |
| 70+ years | 1.0 (0.8-1.2) | 1.2 (0.9-1.5) | 1.3 (1.0-1.8) | 1.4 (1.0-2.1) | 1.5 (1.0-2.4) |  |

NB the smallest 2 symptom groups were excluded from the model due to small numbers and the ‘lump and non-breast’ symptom group was included in the model but is not reported here.

Bold denotes p<0.05. (ref) = reference group

#### Primary care interval

Compared to women with ‘breast lump only’, women with ‘non-breast’ symptoms had particularly long primary care intervals (15-fold greater at the median, p<0.001). Women with ‘non-lump only’ and ‘lump and non-lump’ symptoms also had longer time to referral after adjusting for age group and ethnicity, but this was only seen in the upper quantiles, in other words affecting a smaller number of women.

There was no evidence to support variation in the primary care interval by ethnicity. Younger women (aged <50 years) experienced longer time to referral compared to the reference age group (aged 50–69 years) at the 75^th^ centile and above, but there was no evidence for this at the median.

| **Variable** | **Q(0.25)**  **(95% CI)** | **Q(0.50)**  **(95% CI)** | **Q(0.75)**  **(95% CI)** | **Q(0.90)**  **(95% CI)** | **Q(0.95)**  **(95% CI)** | **Joint Wald test**  **P value** |
| --- | --- | --- | --- | --- | --- | --- |
| Breast lump only (ref) | - | - | - | - | - | <0.001 |
| Non-lump only | 1.0 (1.0-1.0) | 1.0 (1.0-1.0) | **3.0 (1.8-5.0)** | **5.4 (2.7-10.9)** | **4.8 (3.0-7.8)** |  |
| Lump and non-lump | 1.0 (1.0-1.0) | 1.0 (1.0-1.0) | 3.0 (1.0-9.3) | **3.2 (1.1-9.6)** | **7.7 (2.7-22.2)** |  |
| Non-breast symptoms | 1.0 (0.5-2.0) | **15.0**  **(8.1-27.6)** | **57.0**  **(36.3-89.6)** | **41.0**  **(23.8-70.7)** | **25.2**  **(14.3-44.4)** |  |
| White (ref) | - | - | - | - | - | 0.712 |
| Non-white | 1.0 (1.0-1.0) | 1.0 (1.0-1.0) | 1.0 (.7-1.4) | 1.4 (0.6-3.3) | 1.5 (0.6-3.5) |  |
| <50 years | 1.0 (1.0-1.0) | 1.0 (1.0-1.0) | **3.0 (1.5-6.0)** | **3.8 (2.0-7.1)** | **4.4 (2.6-7.6)** | <0.001 |
| 50-69 years (ref) | - | - | - | - | - |  |
| 70+ years | 1.0 (1.0-1.0) | 1.0 (1.0-1.0) | 1.0 (1.0-1.0) | 0.6 (0.3-1.1) | 0.9 (0.5-1.7) |  |

NB the smallest 2 symptom groups were excluded from the model due to small numbers and the ‘lump and non-breast’ symptom group was included in the model but is not reported here.

Bold denotes p<0.05. (ref) = reference group

### Additional references

[1] L.A. Marlow, L.M. McGregor, J.Y. Nazroo, J. Wardle. Facilitators and barriers to help-seeking for breast and cervical cancer symptoms: a qualitative study with an ethnically diverse sample in London. Psychooncology. 23 (2014) 749–57. <http://dx.doi.org/10.1002/pon.3464>

[2] C.C. Burgess, H.W.W. Potts, H Hamed, A.M. Bish, M.S. Hunter, M.A. Richards, et al. Why do older women delay presentation with breast cancer symptoms? Psychooncology. 15 (2006) 962–8. <http://dx.doi.org/10.1002/pon.1030>

[3] H. Jensen, M.L. Tørring, F. Olesen, J. Overgaard, P. Vedsted. Cancer suspicion in general practice, urgent referral and time to diagnosis: a population-based GP survey and registry study. BMC Cancer 14 (2014) 636. <http://dx.doi.org/10.1186/1471-2407-14-636>

[4] H. Jensen, M.L. Tørring, F. Olesen, J. Overgaard, M. Fenger-Grøn, P. Vedsted. Diagnostic intervals before and after implementation of cancer patient pathways–a GP survey and registry based comparison of three cohorts of cancer patients. BMC Cancer 15 (2015) 308. <http://dx.doi.org/10.1186/s12885-015-1317-7>
